# Type 2 Diabetes Remission 5 Years After Laparoscopic Sleeve Gastrectomy: Multicenter Cohort Study

**DOI:** 10.1007/s11695-020-05088-w

**Published:** 2020-11-05

**Authors:** Magdalena Mizera, Michał Wysocki, Katarzyna Bartosiak, Paula Franczak, Hady Razak Hady, Piotr Kalinowski, Piotr Myśliwiec, Michał Orłowski, Rafał Paluszkiewicz, Jerzy Piecuch, Jacek Szeliga, Maciej Walędziak, Piotr Major, Michał Pędziwiatr

**Affiliations:** 1 2nd Department of General Surgery, Jagiellonian University Medical College, Jakubowskiego 2 Street, Building H 3rd floor, 30-688 Kraków, Poland; 2 Department of General, Oncological, Metabolic and Thoracic Surgery, Military Institute of Medicine, Warsaw, Poland; 3 Department of General and Oncological Surgery, Ceynowa Hospital, Wejherowo, Poland; 4 1st Department of General and Endocrin Surgery, Medical University of Białystok, Białystok, Poland; 5 Department of General, Transplant and Liver Surgery, Medical University of Warsaw, Warsaw, Poland; 6 Department of General Surgery, Bariatric Surgery and Emergency Medicine Zabrze, Silesian Medical University, Katowice, Poland; 7 Department of General, Gastroenterological and Oncological Surgery CM, Nicolaus Copernicus University, Toruń, Poland

**Keywords:** Laparoscopic sleeve gastrectomy, Type 2 diabetes mellitus, Diabetes remission

## Abstract

**Purpose:**

Bariatric surgery is no longer considered only as a weight loss surgery but also a way of treating obesity-related comorbidities such as type 2 diabetes mellitus (T2DM). Short-term T2DM remissions in patients undergoing laparoscopic sleeve gastrectomy (LSG) have been shown, but there are very few reports on the mid-term results. We aimed to assess the remission rate of T2DM in obese patients after LSG throughout 5-year follow-up.

**Materials and Methodology:**

We performed a retrospective multicenter cohort analysis of 240 patients who underwent LSG. We assessed the remission rate of T2DM 1 year and 5 years after surgery.

**Results:**

Forty-six percent of patients achieved T2DM remission 5 years after LSG. The remission group had better weight loss results (median% of total weight loss 5 years after: 30.1% (22.9–37.0) vs 23.0% (13.7–30.2), *p* < 0.001) and were significantly younger than the no remission group (43 (38–52) vs 52 (44–58) years, *p* < 0.001). Duration of T2DM was significantly shorter (2 (1–5) vs 5 (3–10) years, *p* < 0.001) with less insulin requirement and less diabetes-related complications (7.2% vs 19.8%, *p* < 0.001) and significantly lower median DiaRem score (4.0 (IQR 2.0–6.0) vs 12.0 (IQR 5.0–16.0), *p* < 0.001). Preoperative body mass index (BMI) had no effect on remission.

**Conclusions:**

Our study suggests that diabetes remission after laparoscopic sleeve gastrectomy occurs frequently, and in the 5-year follow-up, it may remain at the level of 46%. We identified the age of patients, duration, and severity of T2DM as factors affecting mid-term diabetes remission. Nevertheless, further well-designed trials are needed to support our findings.

## Introduction

Obesity is a common health problem worldwide, forming a basis for the occurrence of obesity-related diseases such as type 2 diabetes mellitus (T2DM), hypertension, obstructive sleep apnea syndrome, and even neoplasms [1]. In recent years, the number of bariatric procedures has been growing constantly because they have been proven to be safe and give satisfactory results [2]. Bariatric surgery has been proven beneficial in the treatment of both obesity itself and obesity-related diseases [3]. Laparoscopic sleeve gastrectomy (LSG) has one of the shortest learning curves, which together with good weight loss results explain the fact that it is frequently performed [2, 4]. The remission of obesity-related diseases is now mainly considered to be the reason for the increase in bariatric procedures, and a sole weight loss effect is no longer the only expected result. Bariatric surgery is considered to modulate neural mechanisms, secretion of gastrointestinal hormones, and entail gut microbiota alterations [5]. Many articles have shown the benefits of short-term diabetes remission in patients undergoing LSG, but there are very few reports on the mid-term results [6–8].

## Aim

The aim of this study was to identify the factors affecting mid-term T2DM remission in patients undergoing LSG. We performed a multicenter study to evaluate the effects of LSG on T2DM remission in a 5-year follow-up period.

## Materials and Methods

The study was approved by the institutional review board of the Jagiellonian University Medical College, Krakow, Poland (No. 1072.6120.117.2019). This was a multicenter retrospective observational study. Data from seven Polish surgical centers were collected in a web-based database; each of the centers performs more than 50 LSG procedures a year. Patients were operated on between 2007 and 2014; during this time, 362 LSG procedures were performed in diabetic patients. The LSG technique was comparable in participating centers; the procedure is described elsewhere [9]. Morbidly obese patients with T2DM who underwent LSG and completed at least a 5-year follow-up period were included in the study. Qualification criteria for the bariatric procedure were body mass index (BMI) equal or greater than 40 kg/m^2^ or BMI equal or greater than 35 kg/m^2^ and obesity-related comorbidities [10, 11]. In our analysis, we focused on complete remission of T2DM only. Diagnosis criteria and the status of diabetes after surgery were defined in accordance with the definitions developed by the American Society for Metabolic and Bariatric Surgery (ASMBS) [12]: Complete remission (HbA1c < 6% AND fasting blood glucose < 5.56 mmol/L AND the absence of antidiabetic medications). Very similar definition is proposed by the American Diabetes Association (ADA) [13]: Complete remission (HbA1c < 6% AND fasting blood glucose < 5.56 mmol/L AND no need for antidiabetic drugs for at least 1 year). Unfortunately the definition proposed by ADA cannot be applied in 1-year follow-up, because the time criterion is impossible to fulfill. Nevertheless, in our study in a 5-year follow-up, both definitions can be applied uniformly. Only patients in whom both 1- and 5-year follow-up was obtained were included in the study. Therefore, from the initial number of 362 cases, almost 13% (46 patients) were lost to follow-up in 1 year from the surgery but had available 5-year follow-up results. Additionally 21% (76 patients) of patients were lost to follow-up at 5-year checkup. Therefore, altogether 122 patients (33.7%) were excluded from the analysis. DiaRem score was retrospectively calculated according to Still et al. [14].

The local coordinating surgeons were responsible for data acquisition. The following variables were recorded: annual number of LSGs performed in each participating center; patient characteristics including sex, age, height, maximal body weight, preoperative weight loss, and body weight at the time of surgery and 1 and 5 years after surgery; history of smoking, duration of type 2 diabetes, and status of diabetes one and 5 years after surgery; insulin therapy before and 1 year and 5 years after surgery; other than insulin type of antidiabetic treatment before and 1 year and 5 years after surgery; diabetes-related complications (such as nephropathy, retinopathy, neuropathy, diabetic foot syndrome, and micro- and macroangiopathy); and HbA1c and glycemic values before and 1 year and 5 years after surgery.

Each coordinator at a participating center received a password-protected form, allowing data entry. All data were completely anonymized and did not allow patient identification. This study did not implement any changes in patients’ surgical treatment and perioperative care protocols. For this type of study, formal consent is not required, and informed consent does not apply. The project was supported by the Metabolic and Bariatric Surgery Chapter of the Association of Polish Surgeons.

## Statistical Analysis

Statistical analysis was done using StatSoft Statistica 13.5 PL (StatSoft Inc., Tulsa, OK, USA). Continuous variables were presented as means with standard deviations (SD) or medians with inter-quartile ranges (IQR) for skewed variables. Student’s *t* test for normally distributed variables and the Mann-Whitney test for skewed variables were used to compare the received data. Dichotomous variables were included in Pearson’s chi-square, Yates’, and Fisher’s exact tests, depending on the quantities of data in the subgroups. Categorization of continuous variables was done with the use of ROC analyses. Statistically significant cut-off points of age, duration of T2DM, preoperative HbA1c, %WL 1 year after, HbA1c 1 year after, and %TWL 5 years after were tested in univariate logistic regression models along with other data to determine the factors contributing to diabetes remission. Then, multivariate logistic regression model was built with the use of factors significant in univariate models. No multicollinearity issue was detected in multivariate logistic regression model (VIF < 10 for all variables in the model). Results were considered statistically significant when *p* values were < 0.05.

## Results

Two hundred and forty patients (107 males and 133 females) were included in the study. Median age was 49 years (IQR 40–55), median duration of T2DM before surgery was 4 years (IQR 2–8) (32% 2 years or less, 25% 2–5 years, 20% 5–10 years, and 10% > 10 years; in 13% duration, T2DM is unknown), and median preoperative BMI was 47.6 kg/m^2^ IQR (43.3–52.0). General characteristics of patients are presented in Table 1. Total weight loss (TWL) and metabolic changes in patients with remission after 5 years are presented in Table 2. Patients included were treated with insulin and/or other than insulin type of antidiabetic treatment or with diet only.

**Table 1:** General characteristics of patients

*N* (%)	240
Sex (Male/Female) (%)	107/133 (45%/55%)
Median age, years (IQR)	49 (40–55)
Median maximal preoperative BMI, kg/m^2^ (IQR)	48.7 (43.9–54.2)
Smoking status, n (%)	51 (21.3%)
Median duration of T2DM, years (IQR)	4 (2–8)
Insulin therapy preoperatively, n (%)	71 (29.6%)
Other than insulin type of antidiabetic treatment, *n* (%)	209 (87.1%)
Complications of T2DM, n (%)	33 (13.8%)
Median preoperative HbA1c, % (IQR)*	7.0 (6.2–7.8)
Median preoperative fasting glucose, mmol/L (IQR)*	7.2 (6.1–9.2)

*The data was available only for 175 patients (73%)

**Table 2:** Weight and metabolic changes

Weight and metabolic changes			
After 1 year		After 5 years	
Median BMI 1 year after, kg/m^2^ (IQR)	34.3 (30.8–39.1)	Median BMI 5 years after, kg/m^2^ (IQR)	34.2 (30.8–39.0)
Median %TWL, % (IQR)	26.2 (20.5–32.6)	Median %TWL 5 years after, % (IQR)	26.1 (17.9–34.4)
Median %EWL, % (IQR)	55.4 (43.6–70.0)	Median %EWL 5 years after, % (IQR)	55.8 (38.4–72.6)
Median HbA1c 1 year after, % (IQR)	5.8 (5.3–6.1)	Median HbA1c 5 years after, % (IQR)	5.8 (5.4–6.0)
Median fasting glucose 1 year after, mmol/L (IQR)	5.5 (5.0–6.3)	Median fasting glucose 5 years after, mmol/L (IQR)	5.6(5.1–6.3)
Insulin sc. 1 year after, *n* (%)	46 (19.2%)	Insulin sc. 5 years after, n (%)	29 (12.1%)
Other than insulin type of antidiabetic treatment 1 year after, *n* (%)	104 (43.3%)	Other than insulin type of antidiabetic treatment 5 years after, *n* (%)	101 (42.1%)

Out of the 240 patients in the study, diabetes remission after 5 years was achieved by 111 patients (46.3%), including 92 patients (38.3%) who achieved complete remission 1 year after surgery and maintained diabetes remission for 5 years after surgery and 19 patients (7.9%) who achieved diabetes remission 5 years after surgery. Patients’ remission status after 1 year and 5 years is presented in Table 3.

**Table 3:** Patients remission status after 1 year vs 5 years

	No remission after 5 years	Remission after 5 years	Total
No remission after 1 year	107 (45%)	19 (8%)	126
Remission after 1 year	22 (9%) relapse	92 (38%)	114
Total	129	111	240

Characteristics of patients 5 years after surgery are presented in Table 4. Remission patients were significantly younger than no remission patients (*p* < 0.001) and had a shorter time of duration of diabetes before surgery (*p* < 0.001); the number of diabetes-related complications was significantly smaller in the remission group (*p* = 0.005). Patients who achieved remission had better bariatric effect—greater median %TWL and median %EWL—but the median preoperative BMI did not affect diabetes remission (*p* = 0.814). Weight and metabolic changes of patients with diabetes remission 5 years after the surgery are presented in Table 5.

**Table 4:** General characteristics of patients with diabetes remission 5 years after the surgery

	Remission	No Remission	*p* value
*N* (%)	111	129	n/a
Males/females, *n* (%)	49/62 (44%/56%)	58/71 (45%/55%)	0.899
Median age, years (IQR)	43 (38–52)	52 (44–58)	< 0.001
Median preoperative BMI, kg/m^2^ (IQR)	48.2 (43.6–51.4)	47.3 (43.0–52.2)	0.814
Median preoperative ΔBMI, kg/m^2^ (IQR)	1.0 (0.0–3.0)	0.7 (0–1.8)	0.068
Smoking status, *n* (%)	25.0 (22.5%)	26.0 (20.2%)	0.655
Median duration of T2DM, years (IQR)	2 (1–5)	5 (3–10)	< 0.001
Insulin therapy, n (%)	9.0 (8.1%)	62.0 (48.1%)	< 0.001
Median amount of insulin I.U. /24 h, n (IQR)	45 (27–100)	48 (36–72)	0.881
Other than insulin type of antidiabetic treatment, *n* (%)	92 (82.9%)	117 (90.7%)	0.072
Complications of T2DM, *n* (%)	8 (7.2%)	24 (19.8%)	0.005

**Table 5:** Weight and metabolic changes of patients with diabetes remission 5 years after the surgery

	Remission	No remission	*p* value
Median preoperative HbA1c, % (IQR) *	6.6 (6.2–7.0)	7.2 (6.6–8.2)	< 0.001
Median preoperative fasting glucose, mmol/L (IQR)*	6.4 (5.8–7.6)	7.8 (6.6–10.0)	< 0.001
Median BMI 5 years after, kg/m^2^ (IQR)	32.8 (30.0–36.8)	35.3 (32.1–41.3)	< 0.001
Median %TWL 5 years after, % (IQR)	30.1 (22.9–37.0)	23.0 (13.7–30.2)	< 0.001
Median %EWL 5 years after, % (IQR)	66.6 (47.8–76.6)	47.3 (31.4–67.0)	< 0.001
Median HbA1c 5 years after, % (IQR)	5.4 (5.2–5.8)	5.9 (5.5–6.5)	< 0.001

*The data was available only for 175 patients (73%)

Full data allowing DiaRem score calculation was available for 176 patients (73%). This was due to the fact that the status of diabetes remission was assessed in accordance with the guidelines by the chief surgeon when entering to the database. An exact level of HbA1c could be entered if obtainable, but was not obligatory. Median score for patients with diabetes remission was significantly lower than for patients without remission (4.0 vs 12.0, *p* < 0.001). Patients who achieved diabetes remission scored mostly 3–7 points (52%) or 0–2 points (38%). Patients who did not achieve diabetes remission most frequently scored 13–17 points (40%), 8–12 points (15%), and 3–7 points (22%). Specific DiaRem scores are presented in Table 6.

**Table 6:** DiaRem score results

DiaRem Score	All (*n* = 176)	Remission (*n* = 77)	No remission (*N* = 99)
0–2	42 (24%)	29 (38%)	13 (13%)
3–7	62 (35%)	40 (52%)	22 (22%)
8–12	19 (11%)	4 (5%)	15 (15%)
13–17	44 (25%)	4 (5%)	40 (40%)
18–22	9 (5%)	0	9 (9%)

Univariate logistic regression models demonstrated that younger patients (OR 0.94, 95% CI 0.91–0.96) with shorter median diabetes duration (OR 0.81, 95% CI 0.74–0.88) had a higher chance of diabetes remission. Patients receiving insulin treatment before surgery were less likely to achieve remission (OR 0.10, 95% CI 0.04–0.21). Remission was more likely to occur in patients with fewer diabetes complications (OR 0.31, 95% CI 0.13–0.74) and lower HbA1c (OR 0.58, 95% CI 0.44–0.77) and fasting glucose levels (OR 0.77, 95% CI 0.67–0.88) preoperatively. After 5 years, every 1% TWL accretion and 1% EWL accretion positively impacted diabetes remission, with OR 1.06 (95% CI 1.03–1.08) and OR 1.03 (95% CI 1.02–1.04), respectively. There is an inverse correlation between DiaRem score and the odds ratio for diabetes remission (OR: 0.32; 95% CI 0.23–0.46, *p* < 0.001) in univariate logistic regression model.

The data for the multivariate logistic regression model 5 years after surgery is presented in Table 7. Calculations demonstrated that factors such as age over 45 years, preoperative insulin treatment, other than insulin type of antidiabetic treatment 1 year after surgery, and weight loss percentages 5 years after surgery greater than 22.0% affected the odds ratio of type 2 diabetes mellitus remission 5 years after LSG.

**Table 7:** Multivariate logistic regression model of factors influencing odds ratio of T2DM remission 5 years after LSG

	OR	95%CI	*p* value
Age > 45 years	0.19	0.04–0.81	0.023
Median duration of T2DM > 4 years	1.90	0.39–9.20	0.420
Insulin therapy	0.13	0.02–0.98	0.045
Complications of T2DM	0.58	0.08–4.34	0.589
Preoperative HbA1c > 7.05%	0.61	0.13–2.82	0.525
%TWL 1 year after > 23.78%	1.25	0.32–4.89	0.744
HbA1c 1 year after > 6%	1.25	0.17–9.10	0.822
Insulin sc. 1 year after	0.13	0.01–2.40	0.163
Other than insulin type of antidiabetic treatment 1 year after	0.03	0.01–0.19	< 0.001
%TWL 5 years after > 22.0%	21.20	3.31–135.52	0.001
Category of DiaRem	0.79	0.40–1.56	0.495

## Discussion

This study showed that T2DM remission 5 years after LSG can be observed in almost half of the patients. They had better postoperative weight loss, were younger, and the duration of T2DM was shorter with less insulin requirement and less diabetes-related complications. We also discovered that preoperative BMI had no effect on the remission status.

The number of bariatric procedures performed worldwide is constantly increasing, and recently LSG overtook laparoscopic Roux-en-Y gastric bypass (LRYGB), representing 46.0% and 38.2%, respectively, of all operations performed since 2014 [2]. This trend may be somewhat due to the relatively shorter learning curves and shorter operative time as well as satisfactory clinical outcomes. Weight loss is no longer the only indication for bariatric surgery. Metabolic changes come hand in hand with weight loss, and hence, the resolution of metabolic disorders such as diabetes is possible. Knowledge of factors promoting diabetes remission together with recognition of both short- and mid-term outcomes of different procedures is crucial to enable conscious decision-making [15–19]. The topic of mid-term remissions after LRYGB has already been extensively studied [20, 21]. There are also many research projects investigating short-term outcomes after LSG [6, 22]. Our study is one of the first to analyze mid-term outcomes of diabetes remission in patients undergoing LSG.

Factors promoting T2DM remission were previously studied using short-term follow-up data [23–26], and they coincide with the factors identified in our research. One-year T2DM remission rates differ between authors (Casajoana et al., 53.3%; Stallard et al., 75%; Murphy et al. 72%) [24–26]. Data on mid-term diabetes remission after LSG vary significantly among the studies. Some achieve only 12.0% [27], 19.0% [28] or 23.0% [29] remission rate. Others however reach remission rate of 61.5% [30], 77.0% [31], or even 81.9% [32]. Recently published network meta-analysis on diabetes remission after bariatric surgeries shows that in the follow-up over 3 years of length after LSG, the diabetes remission rate is on the level of 39.9% [33]. Moreover, the latest published systematic review of literature shows that there is no significant difference between diabetes remission in 2- to 5-year follow-up between RYGB and LSG (50% vs 45%, 95% CI (0.94–1.20)) [34]. This data however is hard to interpret due to combining 2- to 5-year follow up in one analysis—overall 5-year follow-up diabetes remission results are sparse. On the other hand, we observed diabetes relapse in a 5-year follow-up in 22 patients (9.2%), where recently published study [35] estimates late relapse risk after LSG at the level of 36%.

There are several potential explanations for this variance of results. Differences may be caused by different trial designs (cohort studies and RCTs), different follow-up lengths (from 2 to 5 years in various combinations), and different diabetes remission definitions (not all authors use the ASMBS or ADA definition; some do not mention the definition that was used). Relatively large differences in sample size may be also observed (majority of studies included less than 50–70 patients) in comparison to our study. Our results regarding mid-term diabetes remission are in line with the few previously published studies but are supported by a significantly larger sample size.

Most, but not all, of the patients who achieved diabetes complete remission 1 year after surgery maintained diabetes remission in the 5-year follow-up. We observed that 22 (9%) of all 240 patients, after achieving remission initially, had a diabetes relapse 5 years after surgery. Similarly, 19 (8%) of all 240 patients did not achieve diabetes remission 1 year after surgery, but in the 5-year follow-up, they proved to be diabetes free. This single observation is strong proof of the need for longer follow-up to attain reliable results.

Diabetes remission rate in our study is higher after 5 years than at 1 year after the surgery. This stays in contrast to other researchers’ results. We speculate that in patients who show persistent weight loss, diabetes remission may occur after 1 year, which is in line with the results of our study.

Mechanisms in which bariatric surgery influences carbohydrate metabolism and determines diabetes status are multifactorial. It is considered that bariatric surgery modulates neural mechanisms (such as vagal afferent activation) and secretion of gastrointestinal and islet hormones (glucagone, glucagone-like peptide-1, peptide YY, cholecystokinin, ghrelin, and others) [5]. These hormones together with adipokines are suspected to be predictors of durable remission and relapse of diabetes after a bariatric procedure [36]. Gut microbiota are considered to be altering insulin resistance and glucose intolerance [37, 38]. In addition, changes in gut microbiota composition have been proven to be associated with bariatric surgery procedures, although results are inconsistent throughout the literature [5]. Serum bile acids levels and composition correlate with metabolic changes after bariatric procedures as well [39].

In our study, we decided to retrospectively calculate DiaRem scale. It was primarily developed to assess diabetes remission possibility after RYGB, but we wanted to check its applicability in LSG patients. We found that DiaRem score < 8 has high predictive value of diabetes remission. In our opinion, it proves that this scale can be successfully used not only in RYGB but also in LSG patients.

This study also has some limitations. Firstly, the studied data were partially retrospective due to the study design. Only patients for whom full follow-up was obtained were included in the study; therefore, 34% of potential study participants were not included. Remaining diabetic patients not included in the study may be the source of bias. Secondly, relatively short duration of T2DM (57% patients had less than 5 years of diabetes duration, and 77% had less than 10-year T2DM history) may be affecting diabetes remission results. Furthermore, it is likely that procedures performed throughout the years varied in different centers, and the lack of uniformity due to the ongoing learning curve may be the source of bias. We did not analyze surgical techniques or the postoperative course; however, it can be assumed that 5-year follow-up allowed short-term confounding factors such as surgical complications to be eliminated.

## Conclusions

Our study suggests that T2DM remission after laparoscopic sleeve gastrectomy done for severe obesity is frequent and may affect as many as 46% of patients with preoperative diabetes 5 years after the surgery. We identified the age of patients, duration, and severity of type 2 diabetes as factors affecting 5-year diabetes remission. Due to the lack of sufficient research into mid-term diabetes remission, further investigation is necessary.

## References

[1] Ma Y, Yang Y, Wang F, Zhang P, Shi C, Zou Y, et al. Obesity and risk of colorectal cancer: a systematic review of prospective studies. Plos one. 2013. 10.1371/journal.pone.0053916 PMC3547959 23349764

[2] Welbourn R, Hollyman M, Kinsman R, Dixon J, Liem R, Ottosson J (2019). Bariatric surgery worldwide: baseline demographic description and one-year outcomes from the fourth IFSO global registry report 2018. Obes Surg.

[3] Hariri K, Guevara D, Dong M, Kini SU, Herron DM, Fernandez-Ranvier G (2018). Is bariatric surgery effective for co-morbidity resolution in the super-obese patients?. Surg Obes Relat Dis.

[4] Różańska-Walędziak AM, Kowalewski PK, Janik MR, Brągoszewski J, Paśnik K, Bednarczyk G (2019). Present trends in bariatric surgery in Poland. Videosurgery Other Miniinvasive Tech.

[5] Cornejo-Pareja I, Clemente-Postigo M, Tinahones FJ. Metabolic and endocrine consequences of bariatric surgery. Front. Endocrinol. (Lausanne). Frontiers Media S.A.; 2019. p. 626. 10.3389/fendo.2019.00626 PMC6761298 31608009

[6] Huang X, Liu T, Zhong M, Cheng Y, Hu S, Liu S (2018). Predictors of glycemic control after sleeve gastrectomy versus Roux-en-Y gastric bypass: a meta-analysis, meta-regression, and systematic review. Surg Obes Relat Dis.

[7] Du X, Zhou HX, Zhang SQ, Tian HM, Zhou ZG, Cheng Z (2017). A comparative study of the metabolic effects of LSG and LRYGB in Chinese diabetes patients with BMI<35 kg/m2. Surg Obes Relat Dis.

[8] Cho JM, Kim HJ, Menzo EL, Park S, Szomstein S, Rosenthal RJ (2015). Effect of sleeve gastrectomy on type 2 diabetes as an alternative treatment modality to Roux-en-Y gastric bypass: systemic review and meta-analysis. Surg Obes Relat Dis.

[9] Major P, Wysocki M, Pędziwiatr M, Pisarska M, Dworak J, Małczak P (2017). Risk factors for complications of laparoscopic sleeve gastrectomy and laparoscopic Roux-en-Y gastric bypass. Int J Surg.

[10] Yumuk V, Tsigos C, Fried M, Schindler K, Busetto L, Micic D, Toplak H, Obesity Management Task Force of the European Association for the Study of Obesity (2015). European guidelines for obesity management in adults. Obes Facts.

[11] Fried M, Yumuk V, Oppert JM, Scopinaro N, Torres A, Weiner R (2014). Interdisciplinary European guidelines on metabolic and bariatric surgery. Obes Surg.

[12] Brethauer SA, Kim J, el Chaar M, Papasavas P, Eisenberg D, Rogers A (2015). Standardized outcomes reporting in metabolic and bariatric surgery. Obes Surg.

[13] Buse JB, Caprio S, Cefalu WT, Ceriello A, Del Prato S, Inzucchi SE (2009). How do we define cure of diabetes?. Diabetes Care.

[14] Still CD, Wood GC, Benotti P, Petrick AT, Gabrielsen J, Strodel WE, Ibele A, Seiler J, Irving BA, Celaya MP, Blackstone R, Gerhard GS, Argyropoulos G (2014). Preoperative prediction of type 2 diabetes remission after Roux-en-Y gastric bypass surgery: a retrospective cohort study. Lancet Diabetes Endocrinol.

[15] Neagoe R, Muresan M, Timofte D, Darie R, Razvan I, Voidazan S, Muresan S, Sala D (2019). Long-term outcomes of laparoscopic sleeve gastrectomy – a single-center prospective observational study. Videosurgery Other Miniinvasive Tech.

[16] Gemici E, Kones O, Seyit H, Surek A, Cikot M, Abdussamet Bozkurt M (2019). Outcomes of laparoscopic sleeve gastrectomy by means of esophageal manometry and pH-metry, before and after surgery. Videosurgery Other Miniinvasive Tech.

[17] Dincer M, Dogan F (2019). The effect of concomitant cholecystectomy and sleeve gastrectomy on morbidity in high-risk obese patients with symptomatic gallstones. Videosurgery Other Miniinvasive Tech.

[18] Wysocki M, Walędziak M, Hady HR, Czerniawski M, Proczko-Stepaniak M, Szymański M (2019). Type 2 diabetes mellitus and preoperative HbA1c level have no consequence on outcomes after laparoscopic sleeve gastrectomy—a cohort study. Obes Surg.

[19] Major P, Wysocki M, Pędziwiatr M, Pisarska M, MaŁczak P, Wierdak M (2018). More stapler firings increase the risk of perioperative morbidity after laparoscopic sleeve gastrectomy. Videosurgery Other Miniinvasive Tech.

[20] Obeid NR, Malick W, Concors SJ, Fielding GA, Kurian MS, Ren-Fielding CJ (2016). Long-term outcomes after Roux-en-Y gastric bypass: 10- to 13-year data. Surg Obes Relat Dis.

[21] Kothari SN, Borgert AJ, Kallies KJ, Baker MT, Grover BT (2017). Long-term (>10-year) outcomes after laparoscopic Roux-en-Y gastric bypass. Surg Obes Relat Dis.

[22] Chouillard EK, Karaa A, Elkhoury M, Greco VJ (2011). Laparoscopic Roux-en-Y gastric bypass versus laparoscopic sleeve gastrectomy for morbid obesity: case-control study. Surg Obes Relat Dis.

[23] Madadi F, Jawad R, Mousati I, Plaeke P, Hubens G. Remission of type 2 diabetes and sleeve gastrectomy in morbid obesity: a comparative systematic review and meta-analysis. Obes. Surg. Springer New York LLC; p. 4066–76. 10.1007/s11695-019-04199-3 31655953

[24] Casajoana A, Pujol J, Garcia A, Elvira J, Virgili N, de Oca FJ, Duran X, Fernández-Veledo S, Vendrell J, Vilarrasa N (2017). Predictive value of gut peptides in T2D remission: randomized controlled trial comparing metabolic gastric bypass, sleeve gastrectomy and greater curvature plication. Obes Surg.

[25] Stallard R, Sahai V, Drover JW, Chun S, Keresztes C (2018). Defining and using preoperative predictors of diabetic remission following bariatric surgery. JPEN J Parenter Enteral Nutr.

[26] Murphy R, Clarke MG, Evennett NJ, John Robinson S, Lee Humphreys M, Hammodat H, Jones B, Kim DD, Cutfield R, Johnson MH, Plank LD, Booth MWC (2018). Laparoscopic sleeve gastrectomy versus banded Roux-en-Y gastric bypass for diabetes and obesity: a prospective randomised double-blind trial. Obes Surg.

[27] Salminen P, Helmiö M, Ovaska J, Juuti A, Leivonen M, Peromaa-Haavisto P, Hurme S, Soinio M, Nuutila P, Victorzon M (2018). Effect of laparoscopic sleeve gastrectomy vs laparoscopic Roux-en-Y gastric bypass on weight loss at 5 years among patients with morbid obesity: the SLEEVEPASS randomized clinical trial. JAMA..

[28] Nedelcu M, Loureiro M, Skalli M, Galtier F, Jaussent A, Deloze M, Gagner M, Fabre JM, Nocca D (2017). Laparoscopic sleeve gastrectomy: effect on long-term remission for morbidly obese patients with type 2 diabetes at 5-year follow up. Surgery..

[29] Schauer PR, Bhatt DL, Kirwan JP, Wolski K, Aminian A, Brethauer SA (2017). Bariatric surgery versus intensive medical therapy for diabetes - 5-year outcomes. N Engl J Med.

[30] Peterli R, Wolnerhanssen BK, Peters T, Vetter D, Kroll D, Borbely Y, et al. Effect of laparoscopic sleeve gastrectomy vs laparoscopic Roux-en-y gastric bypass onweight loss in patients with morbid obesity the sm-boss randomized clinical trial. JAMA - J Am Med Assoc. American Medical Association; 2018;319:255–65. 10.1001/jama.2017.20897 PMC5833546 29340679

[31] Toolabi K, Sarkardeh M, Vasigh M, Golzarand M, Vezvaei P, Kooshki J (2019). Comparison of laparoscopic Roux-en-Y gastric bypass and laparoscopic sleeve gastrectomy on weight loss, weight regain, and remission of comorbidities: a 5 years of follow-up study. Obes Surg.

[32] Ruiz-Tovar J, Carbajo MA, Jimenez JM, Castro MJ, Gonzalez G, Ortiz-de-Solorzano J, Zubiaga L (2019). Long-term follow-up after sleeve gastrectomy versus Roux-en-Y gastric bypass versus one-anastomosis gastric bypass: a prospective randomized comparative study of weight loss and remission of comorbidities. Surg Endosc.

[33] Ding L, Fan Y, Li H, Zhang Y, Qi D, Tang S, Cui J, He Q, Zhuo C, Liu M (2020). Comparative effectiveness of bariatric surgeries in patients with obesity and type 2 diabetes mellitus: a network meta-analysis of randomized controlled trials. Obes Rev.

[34] Borgeraas H, Hofsø D, Hertel JK, Hjelmesæth J (2020). Comparison of the effect of Roux-en-Y gastric bypass and sleeve gastrectomy on remission of type 2 diabetes: a systematic review and meta-analysis of randomized controlled trials. Obes Rev.

[35] Aminian A, Vidal J, Salminen P, Still CD, Hanipah ZN, Sharma G (2020). Late relapse of diabetes after bariatric surgery: not rare, but not a failure. Diabetes Care.

[36] Chen X, Kong X. Diabetes remission and relapse after metabolic surgery. J. Diabetes Investig. Blackwell Publishing; 2018. p. 1237–8. 10.1111/jdi.12871 PMC6215947 29870156

[37] Heianza Y, Sun D, Li X, DiDonato JA, Bray GA, Sacks FM (2019). Gut microbiota metabolites, amino acid metabolites and improvements in insulin sensitivity and glucose metabolism: the POUNDS lost trial. Gut..

[38] Pedersen HK, Gudmundsdottir V, Nielsen HB, Hyotylainen T, Nielsen T, Jensen BAH, Forslund K, Hildebrand F, Prifti E, Falony G, le Chatelier E, Levenez F, Doré J, Mattila I, Plichta DR, Pöhö P, Hellgren LI, Arumugam M, Sunagawa S, Vieira-Silva S, Jørgensen T, Holm JB, Trošt K, Kristiansen K, Brix S, Raes J, Wang J, Hansen T, Bork P, Brunak S, Oresic M, Ehrlich SD, Pedersen O, MetaHIT Consortium (2016). Human gut microbes impact host serum metabolome and insulin sensitivity. Nature..

[39] De Vuono S, Ricci MA, Nulli Migliola E, Monti MC, Morretta E, Boni M (2019). Serum bile acid levels before and after sleeve gastrectomy and their correlation with obesity-related comorbidities. Obes Surg.

